# Combined SUVmax and localized colonic wall thickening parameters to identify high-risk lesions from incidental focal colorectal ^18^F-FDG uptake foci

**DOI:** 10.3389/fonc.2022.972096

**Published:** 2022-08-12

**Authors:** Wenmin Xu, Hansen Li, Ziqian Guo, Linqi Zhang, Rusen Zhang, Long Zhang

**Affiliations:** ^1^ Department of Endoscopy, Affiliated Cancer Hospital and Institute of Guangzhou Medical University, Guangzhou, China; ^2^ Department of Nuclear Medicine, Affiliated Cancer Hospital and Institute of Guangzhou Medical University, Guangzhou, China

**Keywords:** PET/CT, incidental focal colorectal ^18^F-FDG uptake, SUV_max_, CWT, high-risk lesions

## Abstract

**Objective:**

To evaluate the detection ability of ^18^F-FDG PET/CT for identifying high-risk lesions (high-risk adenomas and adenocarcinoma) from incidental focal colorectal ^18^F-FDG uptake foci combining maximum standard uptake value (SUVmax) and localized colonic wall thickening (CWT). The secondary objective was to investigate the factors of missed detection of high-risk adenomas by ^18^F-FDG PET/CT.

**Patients and methods:**

A total of 6394 patients who underwent ^18^F-FDG PET/CT in our hospital from August 2019 to December 2021 were retrospectively analysed, and 145 patients with incidental focal colorectal ^18^F-FDG uptake foci were identified. The optimal cut-off value of SUVmax for ^18^F-FDG PET/CT diagnosis of high-risk lesions was determined by receiver operating characteristic (ROC) curves. SUVmax and localized CWT were combined to identify high-risk lesions from incidental focal colorectal ^18^F-FDG uptake foci. The characteristics of incidental adenomas detected and high-risk adenomas missed by ^18^F-FDG PET/CT were compared.

**Results:**

Of the 6394 patients, 145 patients were found to have incidental focal colorectal FDG uptake foci (2.3%), and 44 patients underwent colonoscopy and pathological examination at the same time. In fact, 45 lesions, including 12 low-risk lesions and 33 high-risk lesions (22 high-risk adenomas, 11 adenocarcinoma), were found by colonoscopy. The area under the ROC curve of SUVmax for low-risk lesions and high-risk lesions was 0.737, and the optimal cut-off value was 6.45 (with a sensitivity of 87.9% and specificity of 58.3%). When SUVmax ≥6.45, the combination of localized CWT parameters has little influence on the sensitivity and specificity of detection; when SUVmax <6.45, the combination of localized CWT parameters can improve the specificity of detection of high-risk lesions, but the sensitivity has little change. In addition, the size of high-risk adenomas discovered incidentally by ^18^F-FDG PET/CT was larger than that of high-risk adenomas missed, but there was no significant difference in lesion location, pathological type or intraepithelial neoplasia between the two groups.

**Conclusions:**

The combination of SUVmax and localized CWT parameters of ^18^F-FDG PET/CT helped identify high-risk lesions from incidental focal colorectal ^18^F-FDG uptake foci, especially for lesions with SUVmax <6.45. Lesion size may be the only factor in ^18^F-FDG PET/CT missing high-risk adenomas.

## Introduction

F-18 fluorodeoxyglucose (^18^F-FDG) positron emission tomography (PET/CT) has become one of the most commonly used positron emission radiography examinations for various oncology imaging, has been used for tumour diagnosis, staging and therapeutic effect prediction and evaluation (1–3), and has been applied to screen second primary cancer in cancer patients (4).

^18^F-FDG PET/CT examination incidentally shows unexpected abnormal areas while performing these functions for patients with noncolorectal indications. Segmental or diffuse uptake of colorectal tissue is often indicative of physiological uptake or inflammatory lesions, while focal FDG uptake is often more important and may indicate colorectal tumours (5–7). Incidental focal colorectal ^18^F-FDG uptake is a relatively rare event, and the incidence of incidental colorectal ^18^F-FDG uptake with PET/CT is approximately 2% (8). Colonoscopy is a powerful method to detect colorectal lesions, and endoscopic resection of adenomatous polyps can reduce the mortality of colorectal cancer (9). However, it is an invasive examination. There are risks of intestinal perforation, intestinal bleeding and anaesthesia (10, 11), and intestinal preparation is troublesome (12). Some frail cancer patients may not be able to tolerate colonoscopy. Whether cancer patients with incidental focal colorectal ^18^F-FDG uptake foci during ^18^F-FDG PET/CT examinations undergo further colonoscopy is still controversial. There is still no consensus on the best strategy. SUVmax represents the maximum standard uptake value of PET during scanning and the quantitative index of the radioactive uptake value of lesions. Increased SUVmax may indicate malignancy. Localized CWT is one of the appearances of the colonic wall on CT of the abdomen, which may also suggest the presence of an underlying neoplasia (13). These two parameters are available in ^18^F-FDG PET/CT. To our knowledge, however, few studies have combined these two parameters to assess whether incidental focal colorectal ^18^F-FDG uptake foci. In this study, we combined SUVmax with localized CWT to distinguish whether incidentally focal colorectal ^18^F-FDG uptake foci are high-risk lesions and should be recommended for further colonoscopy. The secondary objective of this study was to investigate the factors contributing to the missed detection of high-risk adenomas by ^18^F-FDG PET/CT.

## Methods

### Patients and methods

**Figure 1:**
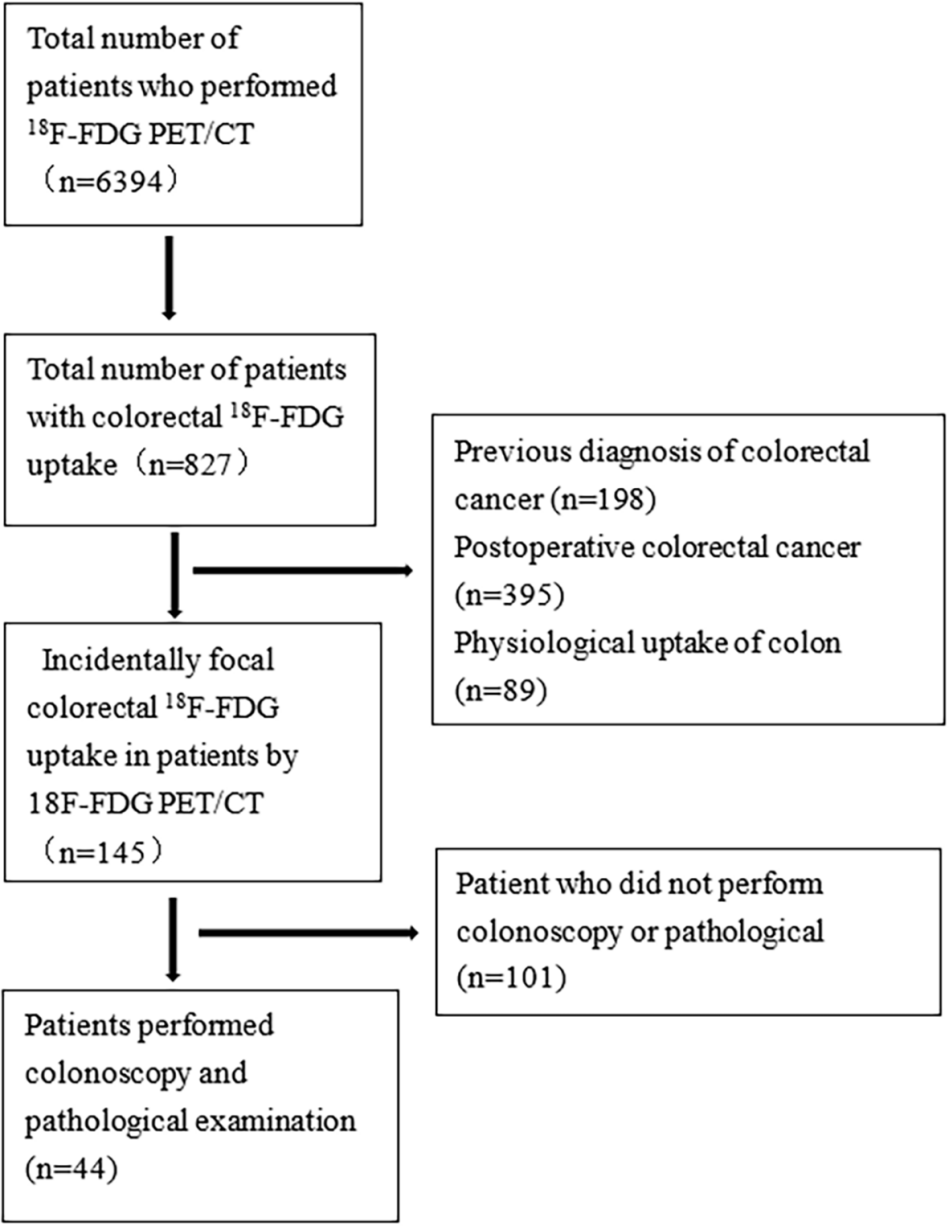
Screening process for patients with incidental focal colorectal ^18^F-FDG uptake foci.

This study is a retrospective study and has been approved by the Institutional Ethics Committee of the Affiliated Cancer Hospital and Institute of Guangzhou Medical University, and the requirement to obtain written informed consent was waived. The information on the patients in our study was mainly obtained from the endoscopy, radiology, and pathology databases of our hospital. The data of patients who underwent ^18^F-FDG PET/CT and colonoscopy from August 2019 to December 2021 were reviewed. During the study, 6394 patients underwent ^18^F-FDG PET/CT at the Affiliated Cancer Hospital and Institute of Guangzhou Medical University. A total of 682 patients were found to have abnormal colorectal ^18^F-FDG uptake on ^18^F-FDG PET/CT examination. Among them, 198 patients diagnosed with colorectal cancer, 395 patients with postoperative colorectal cancer and 89 patients with physiological uptake were excluded. Finally, 145 patients with incidental colorectal ^18^F-FDG uptake foci were found by ^18^F-FDG PET/CT, of whom 44 patients underwent colonoscopy and pathological biopsy examination at the same time (**Figure 1**). High-risk adenomas were defined as those with a diameter greater than or equal to 10 mm, with villous composition (>25.0%) or with high-grade intraepithelial neoplasia or above (14).

### ^18^F-FDG PET/CT protocal

All eligible patients underwent PET/CT scanning (Discovery 710, GE healthcare, Milwaukee, WI, USA) after fasting for at least 6 hours to maintain venous blood glucose levels below 10 mmol/L before administration of ^18^F-FDG. Preexamination blood glucose levels were recorded. Then, patients were intravenously injected with ^18^F-FDG and quietly rested for 60 minutes. CT scan was performed from cranial top to plantar first, with a current of 80-250mA, a total voltage 120kV, a layer thickening of 5.0mm and rotation time of 0.5s; PET image was acquired from cranial top scan to planta for 60-70s per bed and was reconstructed by ordered subset maximum expected value method (OSEM) with 33 subset and 512×512 matrix. Coronal plane, sagittal plane, cross section and maximum density projection image were obtained by CT attenuation correction.

### Image analysis

All data were transferred to the Advantage Workstation (version AW 4.7, GE Healthcare, Milwaukee, WI, USA) and reconstructed using the Bayesian penalized likelihood reconstruction algorithm (Q.clear, GE Healthcare, Milwaukee, WI, USA) with penalization factor (beta) of 500. The semi-automatic quantitative measurements began with manually placement of an oval frame that needed to be drawn to include primary lesions and adjusted to exclude organs with high physiological uptake. SUVmax would be calculated and generated automatically after work frame is placed.

^18^F-FDG PET/CT reports were recorded by junior physicians in the nuclear medicine department and reviewed by experienced ^18^F-FDG PET/CT nuclear medicine specialists. According to the location, shape, size, localized CWT, radioactivity distribution, and SUVmax of the lesions, the characteristic of suspicious colorectal lesions was diagnosed by specialists. Besides, ≥3mm for the colon and ≥5mm for the rectum were considered as increased wall thickness. Localized thickening was defined as CWT that was localized and present in only one of parts of the colon.

### Colonoscopy examination

All eligible patients underwent dietary preparation on a half-flow diet 2 days in advance and then a liquid diet 1 day in advance. Polyethylene glycol electrolytes (PGE; 137.15g; Shenzhen Wanhe Pharmaceutical Co., Ltd, Shenzhen, Guangdong Province, China) dissolved with 2000mL water was administered for bowel preparation six hours before colonoscopy examinations. Complete colonoscopy (Olympus CV-290, Olympus Medical Systems Corp, Tokyo, Japan) from caecal insertion to withdrawal of the colonoscopy was perform using the LCI or WL in all patients. The bowel cleanliness score (Boston score) was ≥7 in all patients (15). If colorectal neoplasms were found during the inspection, their size and location in the colon were estimated by the endoscopic physicians. All data were transferred to the Annet Medical Image Workstation (AnnetWS, Shenzhen Annet Information System Co., Ltd, Shenzhen, Guangdong Province, China). Endoscopic biopsy or electric resection was performed for pathological analysis.

### Data collection and analysis

The statistical description of numerical variable data is expressed as the mean (x ± s), and the statistical description of categorical variable data is expressed as the constituent ratio (%). T test or Mann–Whitney U test was used for statistical data, and Chi-square test or Fisher’s exact probability method was used for statistical data. We applied ROC curves, showing sensitivity and specificity, to evaluate the optimal cut-off value for SUVmax in differentiating colorectal high-risk lesions. *P*<0.05 was considered statistically significant. SPSS 16.0 software (Chicago, IL, United States) was used in the statistical process. GraphPad Prism 8.0.2 software was used for the mapping.

## Results

### Basic patient characteristics

**Figure 2:**
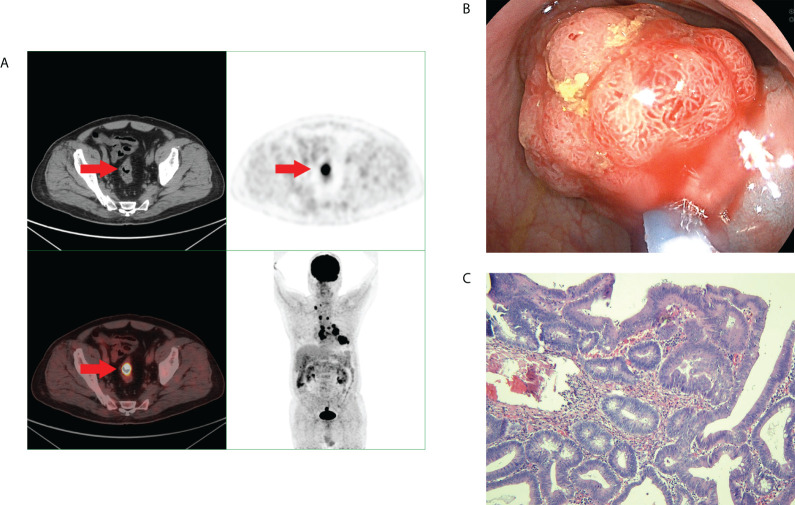
A middle-aged man underwent 18F-FDG PET/CT for left lung squamous cell carcinoma. Incidental focal 18F-FDG uptake was found in thesigmoid colon. The patient underwent colonoscopy and pathology examination. **(A)** Abnormal 18F-FDG PET/CT uptake in the sigmoid colon. **(B)** shows a sigmoid colonpolyp with a diameter of 2.5 cm. **(C)** shows the pathological pattern of villous tubular adenoma with high-grade intraepithelial neoplasia.

**Figure 3:**
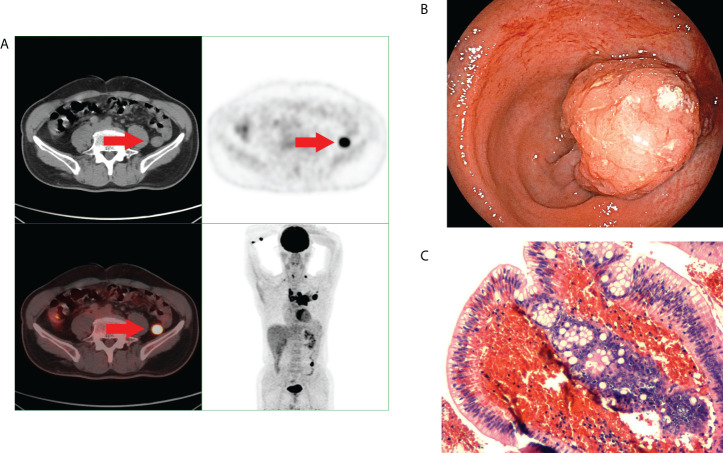
A middle-aged man underwent 18F-FDG PET/CT for left pulmonary nodules. Incidental focal uptake of 18F-FDG was found in the rectum. The patient underwent colonoscopy and pathology examination. **(A)** Abnormal uptake of 18F-FDG PET/CT in the rectum. **(B)** shows a colonoscopic pedicled polyp with a diameter of approximately 2.0 cm. **(C)** shows the pathological type of villous tubular adenoma.

^18^F-FDG PET/CT found 44 patients with incidental focal colorectal FDG uptake foci (**Figures 2**, **3**). Among the 44 patients with incidental focal colorectal ^18^F-FDG uptake, there were 32 males and 12 females, with an average age of 62.12 ± 9.96 years (37-78 years), and the top three indications of ^18^F-FDG PET/CT examination were lung cancer or lung nodule or lung cancer after surgery (18.2%), malignant liver tumour (13.6%) and metastatic cancer with unknown primary foci (9.1%). (**Table 1**).

**Table 1:** Basic information of patients with incidental focal colorectal ^18^F-FDG uptake foci (*n*=44).

Basic information	Average value/number
Age ( ± s, years):	62.12 ± 9.96
Sex[n(%)]: Male Female	32 (72.7) 12 (27.3)
Diagnosis [n (%)]	
Nasopharyngeal carcinoma	3 (6.8)
Lung cancer or lung nodule or lung cancer after surgery	8 (18.2)
Liver malignant tumor	6 (13.6)
Cholangiocarcinoma	1 (2.3)
Cervical cancer	3 (6.8)
Melanoma	1 (2.3)
Lymphoma	2 (4.5)
Prostate cancer	1 (2.3)
Breast cancer	1 (2.3)
Kidney cancer	1 (2.3)
Esophageal cancer	4 (9.1)
Abdominal pain in dispute	6 (13.6)
Uterine cancer	2 (4.5)
Metastatic cancer with unknown primary foci	4 (9.1)
Physical examination	1 (2.3)

### SUVmax in identifying high-risk lesions

**Figure 4:**
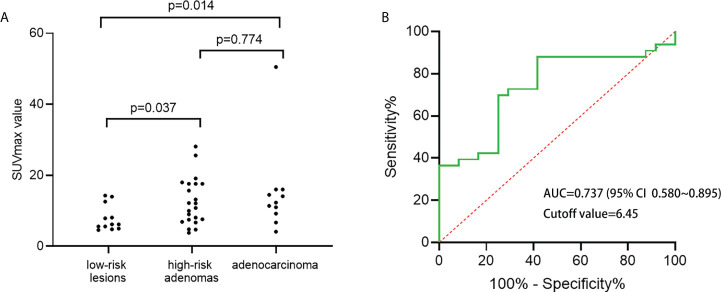
**(A)** SUVmax of low-risk, high-risk adenomas, adenocarcinoma were compared; **(B)** ROC curve of maximum standardized uptake value (SUVmax) for high-risk lesions.

Among the 44 patients with focal incidental colorectal ^18^F-FDG uptake foci, 45 lesions were detected by ^18^F-FDG PET/CT, and 34 lesions were confirmed to be true positive by colonoscopy and pathological results, with a positive predictive value of 75.56%. Among them, low-risk adenoma was found in one patient (2.2%), high-risk adenomas in 22 patients (49.0%) and adenocarcinoma in 11 patients (24.4%). No lesions or inflammatory lesions were found in 11 patients (24.4%) (**Table 2**). We classified normal or inflammatory lesions and low-risk adenoma into one group (low-risk lesion group) and found that the SUVmax in the low-risk lesion group was lower than that in the high-risk adenoma group and adenocarcinoma group (*P*<0.05). However, there was no significant difference in SUVmax between the high-risk adenoma group and the adenocarcinoma group (*P*=0.774) (**Figure 4A**). From the perspective of clinical practice, both high-risk adenomas and adenocarcinoma need to be actively treated. High-risk adenomas and adenocarcinoma with no statistically significant difference in SUVmax were classified into another group (high-risk lesions group). The optimal cut-off point of SUVmax obtained by ROC to distinguish the high-risk lesion group from the low-risk lesion group was 6.45 (**Figure 4B**). When SUVmax=6.45, the sensitivity and specificity were 87.9% and 58.3%, respectively.

**Table 2:** Actual lesions found by colonoscopy (*n*=45).

Lesions classification	The lesion found by colonoscopy:	n (%)
Low-risk lesions	Normal or inflammatory lesions	11 (24.4)
	Low-risk adenoma	1 (2.2)
High-risk lesions	Advanced adenoma	22 (49.0)
	Adenocarcinoma	11 (24.4)

### SUVmax and localized CWT in identifying high-risk lesions

Among the 45 lesions found by 18F-FDG PET/CT, 38 lesions were observed with localized CWT under ^18^F-FDG PET/CT scan, and 7 lesions were not. Localized CWT was observed on ^18^F-FDG PET/CT in 58.3% of low-risk lesions, 90.9% of high-risk adenomas and 100% of adenocarcinomas. There was a difference in the localized CWT between the low-risk lesion group and the high-risk lesion group (*P*=0.010). With localized CWT as the only judgement indicator, it was found that the sensitivity and specificity of the high-risk lesion group were 93.9% and 41.7%, respectively (**Table 3**). We found that when SUVmax ≥6.45 was combined with localized CWT to identify high-risk lesions, the sensitivity or specificity of high-risk lesions did not increase significantly. However, when SUVmax <6.45 was combined with CWT, the specificity of finding high-risk lesions could be improved, while the sensitivity showed little change (**Table 4**).

**Table 3:** Localized colonic wall thickening shown by ^18^F-FDG PET/CT (*n*=45).

	Colonic wall thickening	*P* value
	No Yes	
Low-risk lesions [n (%)]	5(41.7) 7(58.3)	0.010
High-risk lesions [n (%)]	2(6.1) 31(93.9)	

**Table 4:** Sensitivity and specificity of SUVmax and SUVmax combined with localized CWT in the identification of high-risk lesions from incidental focal colorectal ^18^F-FDG uptake foci.

Measures	SUV≥4	SUV≥5	SUV≥6	SUV≥6.45	SUV≥7	SUV≥8	SUV≥9	SUV≥10
Sensitivity (%)	97.0	87.9	87.9	87.9	78.8	72.7	69.7	63.6
Specificity (%)	0.0	16.7	41.7	58.3	58.3	66.7	75.0	75.0
Sensitivity combined with CWT (%)	90.0	87.9	87.9	87.9	78.8	72.7	69.7	63.6
Specificity combined with CWT (%)	50.0	50.0	58.3	66.7	66.7	66.7	75.0	75.0

### Factors of missing high-risk adenomas by ^18^F-FDG PET/CT

In this study, 22 high-risk adenomas were incidentally found by ^18^F-FDG PET/CT. In addition, we searched and compared all patients who underwent ^18^F-FDG PET/CT and colonoscopy at the same time and found that 15 high-risk adenomas were missed by ^18^F-FDG PET/CT. We compared the characteristics of incidental high-risk adenomas and high-risk adenomas missed by ^18^F-FDG PET/CT. The volume of high-risk adenomas missed by ^18^F-FDG PET/CT was smaller than that of incidentally high-risk adenomas missed by ^18^F-FDG PET/CT (*P*=0.001), but there were no significant differences in lesion location, pathological type or intraepithelial neoplasia grade between the two groups (*P*>0.05) (**Table 5**).

**Table 5:** Factors influencing the detection of high-risk adenomas by ^18^F-FDG PET/CT.

	Whether PET/CT shows high risk adenoma FDG uptake		
	No(*n*=15)	Yes(*n*=22)	*P* value
Location [n (%)] Left colon Right colon	10 (33.3) 5 (71.4)	20 (66.7) 2 (28.6)	0.095
Size of neoplasm [n (%)] 5-9mm 10-20mm ≥20mm	8 (80.0) 7 (41.2) 0 (0.0)	2 (20.0) 10 (58.8) 10 (90.9)	0.001
Pathology [n (%)] Tubular adenoma Tubular villous adenoma	5 (50.0) 10 (37.0)	5 (50.0) 17 (63.0)	0.708
Intraepithelial neoplasia [n (%)] Non Low grade intraepithelial High grade intraepithelial or carcinoma in sit	1 (20.0) 7 (53.8) 7 (36.5)	4 (80.0) 6 (46.2) 12 (63.2)	0.374

## Discussion

^18^F-FDG PET/CT has been widely used in radiation oncology. Incidental focal colorectal ^18^F-FDG uptake foci, although relatively rare, can alter the management and prognosis of some cancer patients (16). The incidence of incidental focal colorectal ^18^F-FDG uptake was 2.3% in our study, which was similar to previous studies (17, 18). Most studies have recommended further performance of colonoscopy in patients with incidental focal colorectal ^18^F-FDG uptake in ^18^F-FDG PET/CT examination, since a significant proportion of incidental lesions were confirmed to be cancerous or precancerous (17, 19). In our study, it was also confirmed by colonoscopy and pathology that 24.4% of incidental focal colorectal ^18^F-FDG uptake foci were adenocarcinoma and 48.9% were high-risk adenomas, but there were still a considerable proportion of normal or inflammatory lesions (24.4%) and low-risk adenomas (2.2%). Indiscriminate colonoscopy is not beneficial for cancer patients with limited survival or physical examination.

Advanced adenomas have a higher risk of developing into cancer than nonadvanced adenomas (20). A meta-analysis showed a significantly higher incidence of high-risk adenomas compared with patients without adenomas. In addition, colorectal cancer-related mortality was significantly higher in patients with high-risk adenomas than in patients with low-risk adenomas and those without adenomas (21). Therefore, advanced adenomas should also be treated promptly. We classified high-risk adenomas and adenocarcinoma into the high-risk lesion group and differentiated high-risk and low-risk lesions by relevant parameters of ^18^F-FDG PET/CT. Previous studies have suggested that optimal cut-off points for SUVmax can be used to distinguish malignant lesions or high-grade intraepithelial neoplasia (22) or that metabolic volume (MV) and SUVmax can be used to progressively distinguish malignant and atypical hyperplasia from benign colorectal ^18^F-FDG uptake lesions by ^18^F-FDG PET/CT (23). Some studies found that SUVmax could distinguish malignant lesions from other types of lesions but could not distinguish benign lesions from adenomas (24). Different from previous studies, our study evaluated the ability of ^18^F-FDG PET/CT to diagnose incidental high-risk lesions by using SUVmax and localized CWT parameters and found that SUVmax=6.45 was the optimal cut-off value, with a sensitivity of 87.9% and specificity of 58.3%. When lesion SUVmax <6.45 was combined with CWT parameters, the specificity of diagnosis of high-risk lesions was improved, and the sensitivity was not significantly decreased.

Localized CWT may be a predictor of colon cancer, which can be obtained directly from ^18^F-FDG PET/CT. In Bas et al.’s study, 132 patients were found to have CWT by CT and underwent colonoscopy. A total of 28.8% of the patients were found to have malignant tumours, and 22.7% were found to have colorectal polyps, indicating that CWT may indicate malignant tumours. However, a deficiency of this study is the lack of specific data on colonic wall thickening (25). Karacin et al. compensated for this limitation by retrospectively analysing 5300 colonoscopy reports, including 122 patients with CWT, and grading them according to the specific thickness of CWT. Multivariate analysis found that moderate to severe (≥12 mm), focal or asymmetric CWT was a risk factor for cancer (26). In this study, CWT was used as the evaluation parameter of benign and malignant lesions of incidental colorectal FDG uptake to improve the specificity of lesions with SUVmax <6.45. To our knowledge, this is the first time that a combination of SUVmax and localized CWT parameters has been used to evaluate lesions of incidental focal colorectal ^18^F-FDG uptake foci.

In addition, patients who underwent ^18^F-FDG PET/CT and colonoscopy during the same period were analysed, and 34 high-risk adenomas were missed. We compared the pathological characteristics of incidental and missed high-risk adenomas and found that there was a difference in size between the two groups (*P*=0.01). A total of 58.8% of high-risk adenomas with diameters ≥1 cm and 90.9% of high-risk adenomas with diameters ≥2 cm could be detected by ^18^F-FDG PET/CT, while there were no significant differences in lesion location, pathological type or grade of intraepithelial neoplasia between the two groups. The above data suggest that the lesion size may be a decisive factor affecting the detection of high-risk adenomas by ^18^F-FDG PET/CT. We believe that this may be due to the small size (small number of cells) of advanced adenomas and the relatively low degree of cell malignancy compared with malignant tumours, so it cannot be shown on ^18^F-FDG PET/CT. Our results are similar to those of earlier studies that indicated that size is the only variable affecting colorectal tumour detection (27, 28). However, in recent years, some studies have challenged this view. Through univariate and multivariate analysis, Ravizza et al. found that size >10 mm, villus content and a high degree of atypical hyperplasia were all factors affecting PET/CT detection of adenoma (29). In addition to pathological grade and lesion size, flat shape and lesions located near the colon were also correlated with low sensitivity of PET/CT for colorectal adenoma (30). In view of the relatively small number of patients in this study and retrospective study, further discussion and confirmation are still needed in the future.

On the whole, there are several novelties could be highlighted in this study: Firstly, we attempted to provide clinicians with some help in evaluating whether the foci of incidental focal colorectal uptake are high-risk lesions and whether colonoscopy need to be performed actively by these two parameters (SUVmax and localized CWT) of ^18^F-FDG PET/CT. Previous studies have used SUVmax of ^18^F-FDG PET/CT to distinguish benign lesions from malignant lesions (31, 32). However, there may be some limitations to the studies: In Luboldt et al.’s and Putora et al.’s study, they concluded that SUVmax ≥ cut-off value, patients should be considered for colonoscopy, when SUVmax < cut-off value, they still requires individual evaluation or can’t rule out malignant lesion (33, 34). CWT is a condition encountered on computed tomography, which may be associated with colonic malignancy, inflammation or other benign lesions (13, 25). Further research found that localized CWT may be more associated with malignancy (26, 35). The results of our study showed that when SUVmax < 6.45 (cut-off value), the combination of localized CWT parameters could improve the specificity of the diagnosis of high-risk lesions, and the sensitivity was not significantly reduced. To our knowledge, few studies have used these two parameters to identify incidental focal colorectal ^18^F-FDG uptake foci as high-risk lesions; Secondly, different from previous study (24), adenomas in this study were subdivided into high-risk adenomas and low-risk adenomas, and high-risk adenomas and adenocarcinoma were included in high-risk lesions, which was more consistent with the necessity of colonoscopy for cancer patients. Thirdly, these two parameters can be easily obtained in ^18^F-FDG PET/CT without obtaining other results such as serological tests of the patient (36). Finally, the influence factor of high-risk adenomas missed by ^18^F-FDG PET/CT is still controversial. Our results found that size may be the main factor affecting missed diagnosis, which may provide a perspective for this controversial point.

The limitations of this study are as follows: it was a single-centre retrospective study, and some ^18^F-FDG PET/CT parameters and clinical information were lacking or difficult to trace. Due to incomplete data, ^18^F-FDG PET/CT only recorded the localized CWT and failed to obtain complete specific values and explore them. Due to the defect of description, no comparative analysis was performed on the morphology of colorectal adenomas when comparing incidental and missed high-risk adenomas.

In conclusion, the combination of SUVmax and localized CWT parameters of ^18^F-FDG PET/CT can help improve the specificity of identifying high-risk lesions from incidental focal colorectal ^18^F-FDG uptake foci, especially for lesions with SUVmax < 6.45. For whether patients with incidental focal colorectal ^18^F-FDG uptake foci need colonoscopy, these two easily available parameters may help clinicians make a strategy. In addition, clinicians should pay attention to the fact that high-risk adenomas may be missed by ^18^F-FDG PET/CT due to small size.

## Data availability statement

The datasets generated for this study are available on reasonable request to the corresponding author.

## Ethics statement

The current study was approved by the Institutional Ethics Committee of the Affiliated Cancer Hospital & Institute of Guangzhou Medical University (No.ZN2022-21), and the need of signed informed consent was waived.

## Author contributions

WX and HL participated in the design of the study and drafted the manuscript. ZG and WX collected the patients’ data and performed analysis. LiZ and RZ offered advice and revised the manuscript. LoZ conceived the study and supervised the project. All authors contributed to the article and approved the submitted version.

## Funding

The publication fees of this study will be funded by Affiliated Cancer Hospital and Institute of Guangzhou Medical University

## Conflict of interest

The authors declare that the research was conducted in the absence of any commercial or financial relationships that could be construed as a potential conflict of interest.

## Publisher’s note

All claims expressed in this article are solely those of the authors and do not necessarily represent those of their affiliated organizations, or those of the publisher, the editors and the reviewers. Any product that may be evaluated in this article, or claim that may be made by its manufacturer, is not guaranteed or endorsed by the publisher.
